# Astaxanthin-loaded polymer-lipid hybrid nanoparticles (ATX-LPN): assessment of potential otoprotective effects

**DOI:** 10.1186/s12951-020-00600-x

**Published:** 2020-03-19

**Authors:** Jiayi Gu, Yuming Chen, Ling Tong, Xueling Wang, Dehong Yu, Hao Wu

**Affiliations:** 1 Department of Otolaryngology-Head and Neck Surgery, Shanghai Ninth People’s Hospital, Shanghai Jiao Tong University, School of Medicine, Shanghai, 200011 China; 2 Ear Institute, Shanghai Jiao Tong University, School of Medicine, Shanghai, 200011 China; 3 Shanghai Key Laboratory of Translational Medicine On Ear and Nose Diseases (14DZ2260300), Shanghai, 200011 China

**Keywords:** Lipid-polymer hybrid nanoparticles, Astaxanthin, Cisplatin-induced ototoxicity, Reactive oxygen species

## Abstract

**Background:**

Ototoxicity is one of the major side effects of platinum-based chemotherapy, especially cisplatin therapy. To date, no FDA approved agents to alleviate or prevent this ototoxicity are available. However, ototoxicity is generally believed to be produced by excessive generation of reactive oxygen species (ROS) in the inner ear, thus leading to the development of various antioxidants, which act as otoprotective agents. Astaxanthin (ATX) is an interesting candidate in the development of new therapies for preventing and treating oxidative stress-related pathologies, owing to its unique antioxidant capacity.

**Methods and results:**

In this study, we aimed to evaluate the potential antioxidant properties of ATX in the inner ear by using the HEI-OC1 cell line, zebrafish, and guinea pigs. Because ATX has poor solubility and cannot pass through round window membranes (RWM), we established lipid-polymer hybrid nanoparticles (LPN) for loading ATX. The LPN enabled ATX to penetrate RWM and maintain concentrations in the perilymph in the inner ear for 24 h after a single injection. ATX-LPN were found to have favorable biocompatibility and to strongly affect cisplatin-induced generation of ROS, on the basis of DCFHDA staining in HEI-OC1 cells. JC-1 and MitoTracker Green staining suggested that ATX-LPN successfully reversed the decrease in mitochondrial membrane potential induced by cisplatin in vitro and rescued cells from early stages of apoptosis, as demonstrated by FACS stained with Annexin V-FITC/PI. Moreover, ATX-LPN successfully attenuated OHC losses in cultured organ of Corti and animal models (zebrafish and guinea pigs) in vivo. In investigating the protective mechanism of ATX-LPN, we found that ATX-LPN decreased the expression of pro-apoptotic proteins (caspase 3/9 and cytochrome-c) and increased expression of the anti-apoptotic protein Bcl-2. In addition, the activation of JNK induced by CDDP was up-regulated and then decreased after the administration of ATX-LPN, while P38 stayed unchanged.

**Conclusions:**

To best of our knowledge, this is first study concluded that ATX-LPN as a new therapeutic agent for the prevention of cisplatin-induced ototoxicity.
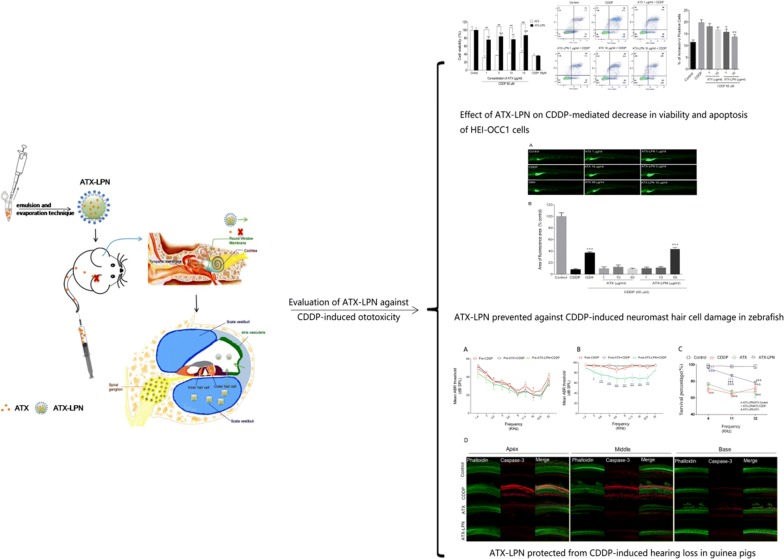

## Background

Cisplatin (cis-diamminedichloroplatinum) (CDDP) is a widely used and highly effective antineoplastic agent for treating various tumors. Unfortunately, CDDP treatment is associated with a high incidence of severe ototoxicity. A recent study has reported that 80% of CDDP-treated patients (388 of 488) experience hearing loss of at least 20 dB, and 40% experience tinnitus [1]. The incidence of CDDP-induced ototoxicity in children ranges from 22 to 77% [2], among which 63%–77% of children experience permanent sensorineural hearing loss [3, 4]. Children are at greater risk of CDDP-induced ototoxicity than adults, thus resulting in severe negative consequences in their neurocognitive ability and psychosocial development [5]. Thus, exploring new agents or strategies to combat the ototoxicity induced by CDDP is crucial.

Although no effective compounds for treatment or prevention of CDDP-induced ototoxicity have been approved by the FDA to date, numerous candidates have been identified in preclinical studies, and some are currently undergoing clinical trials, including antioxidants (dexamethasone, sodium thiosulfate, and acetylcysteine) [6] and anti-inflammatory compounds (Flunarizine and Etanercept) [7]. The molecular mechanism of ototoxicity is not fully understood; however, common characteristics have been observed, such as accumulation of reactive oxygen species (ROS) in hair cells as a result of mitochondrial insult and the release of redox metals interacting with oxygen [8, 9]. Antioxidants have generally been accepted as the most effectively otoprotective agents to combat ototoxicity induced by CDDP.

Astaxanthin (ATX) is widely used as a nutritional supplement and a cosmetic ingredient, owing to its ability to scavenge singlet oxygen and free radicals, and to prevent lipid peroxidation in biological membranes [10, 11]. In recent years, ATX has attracted substantial attention for its unique antioxidant capacity, which is superior to those of vitamin E, beta-carotene, and coenzyme Q10 [12]. The administration of ATX has been shown to effectively prevent the pathogenesis of ROS-related conditions, such as ischemia-related injury in brain tissue [12], neurodegenerative disease such as Alzheimer’s disease [13], cardiovascular diseases and conditions such as ischemia and reperfusion injury [14]. However, to our knowledge, ATX administration in ROS-induced inner ear disease has not been investigated in previous studies.

The present study aimed to evaluate the protective effect of ATX against CDDP-induced ototoxicity in the HEI-OC1 cell line, zebrafish, and guinea pigs, and to explore the underlying molecular mechanisms. The high lipophilicity and thermolability of ATX limits its antioxidant efficacy in humans. To overcome the limitations associated with the inner ear application of ATX noted above, we developed a formulation of lipid-polymer nanoparticle (LPN) carriers to protect ATX’s antioxidant activity and allow for both homogenous dispersion in aqueous solution and strong interactions with hair cells via electrostatic effects. We subsequently evaluated the potential of the resultant LPN carriers in treating CDDP-induced ototoxicity in the HEI-OC1 cell line, zebrafish, and guinea pig animal models.

## Results

### Characterization of ATX-LPN

**Fig. 1:**
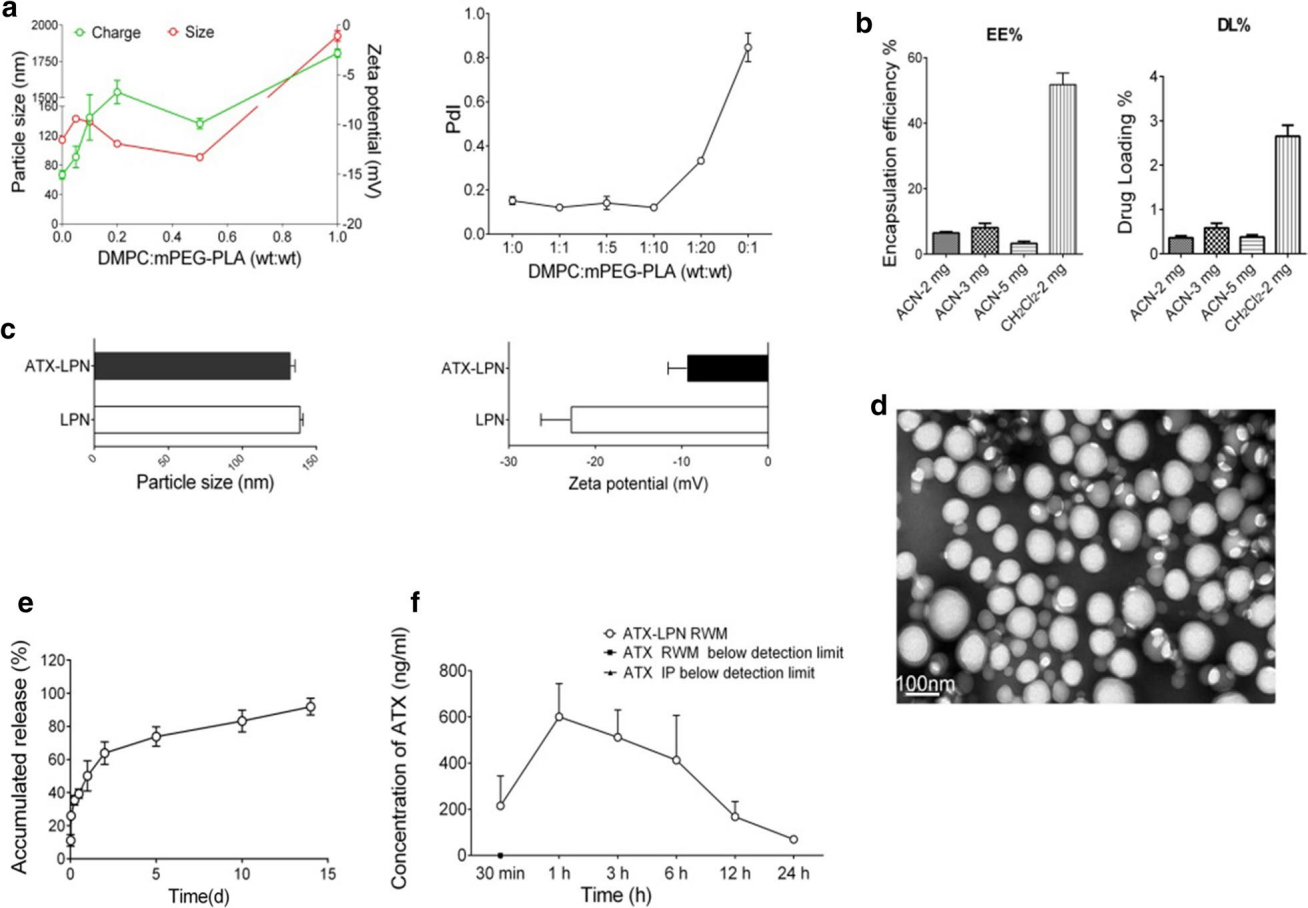
Characterization of ATX-LPN. **a** Particle size (red line), zeta potential (green line) and values of PDI under various ratio of DMPC: mPEG-PLA. **b** Encapsulation efficiency (EE%) and drug loading (DL%) of the ATX-LPN in acetonitrile solution (ACN) or dichloromethane solution (CH_2_CL_2_). **c** Particle size and zeta potential without and with ATX. d Representative TEM images of ATX-LPN. **e** ATX release from ATX-LPN in vitro. **f** ATX-LPN release profile in cochlea perilymph

ATX-LPN were fabricated with an emulsion and evaporation technique and characterized on the basis of particle size, PDI, zeta potential, drug loading (DL)%, and encapsulation efficiency (EE)%. When the ratio of DMPC/mPEG-PLA reached 1:2, the particle size was at a minimum (90.76 ± 0.53 nm) (Fig. 1a), and the value of PDI (0.333 ± 0.003) suggested good dispersity level of the nanoparticles. The diameter of the nanoparticles increased when the content of DMPC either increased or decreased. Specifically, nanoparticles fabricated with mPEG-PLA or DMPC alone were 114.23 ± 1.50 nm and 1925.33 ± 20.84 nm in diameter, respectively. The zeta potential, as well as PDI, increased with the elevation of DMPC content, but all were negatively charged, ranging from − 3 mV to − 15 mV (Fig. 1a). For favorable unifomity and an acceptable size, we selected the ratio of DMPC/mPEG-PLA (1:9) for the following study.

Figure 1b showed that DL % and EE % were much lower in acetonitrile solution at various concentrations than in dichloromethane solution (CH_2_CL_2_), in agreement with ATX’s being more soluble in CH_2_CL_2_ than in acetonitrile. Consequently, we used an emulsion and evaporation technique instead of a nanoprecipitation method. After loading of the drug, the nanoparticle size slightly decreased, from 138.97 ± 0.87 nm to 132.28 ± 1.37 nm (Fig. 1c), whereas the zeta potential greatly decreased, from − 22.77 ± 1.43 to − 9.30 ± 0.93 (Fig. 1c), probably because of the drugs attached to the surfaces of the nanoparticles. The representative transmission electron microscopy images of ATX-LPN revealed that the nanoparticles were spherical with smooth surfaces, and distributed uniformly without adhesion or aggregation (Fig. 1d).

### In vitro and in vivo drug release profiles of ATX-LPN

The release profile of ATX from ATX-LPN in AP is shown in Fig. 1e. The release profile showed an initial burst release of ATX during the first 24 h, in which approximately 50.09% of the encapsulated ATX was released from LPN. Subsequently, the rate of ATX release slowed down. A sustained release pattern was observed from 24 h to 15 d. At the end of the 15 d release, the cumulative release of ATX from LPN was 91.90%.

Next, we detected the ATX concentrations in cochlear perilymph in vivo at various time points (0.5, 1, 3, 6, 12, and 24 h after RWM administration of ATX/ATX-LPN. As shown in Fig. 1f, the ATX concentration was below the detection limit, thus indicating that ATX could not pass through the RWM when administered by IP or RWM. However, 30 min after ATX-LPN were administered through the RWM, the ATX concentration was detected to be 214.88 ± 129.47 ng/ml. The ATX concentration peaked at 1 h (600.67 ± 143.16 ng/ml) and gradually decreased during the post-treatment time. ATX remained detectable after 24 h (69.91 ± 13.67 ng/ml). The ATX release lasted for 24 h in cochlear perilymph in the inner ear. These results indicated that ATX-LPN penetrated through the RWM and showed a long-term drug release profile, which may be beneficial for inner ear administration.

### Cellular uptake of coumarin 6-LPN

**Fig. 2:**
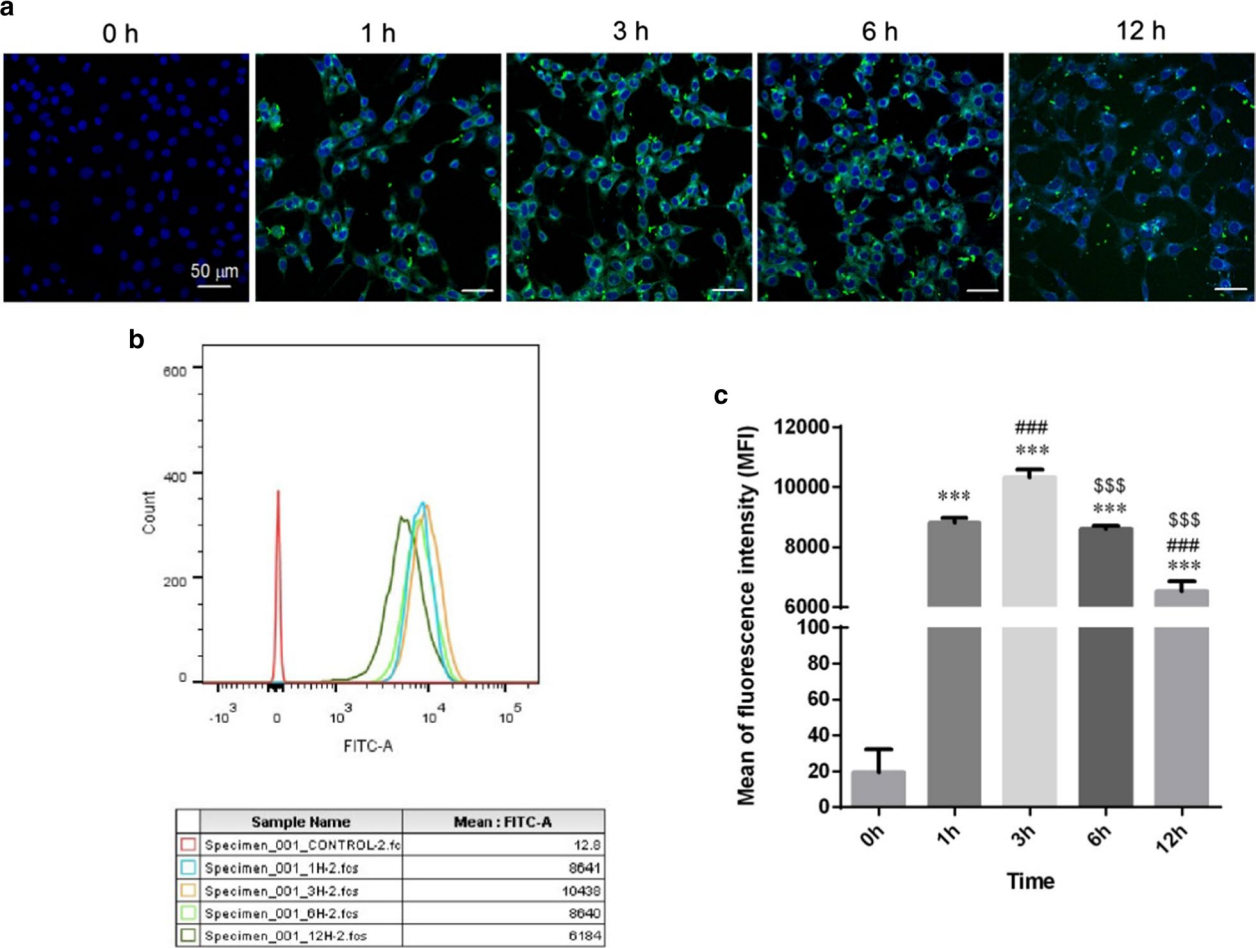
Cellular uptake of coumarin 6-LPN on HEI-OC1 cells. **a** confocol images of internalization at different times. **b** Flow cytometry assay. **c** Mean fluorescence intensity of HEI-OC1 cells by flow cytometry assay. ***p < 0.001 as compared with 0 h, ^###^p < 0.001 as compared with 1 h, ^$$$^p < 0.001 as compared with 3 h

Cellular uptake of coumarin 6-LPN in HEI-OC1 cells was examined through both confocal laser scanning microscopy and flow cytometry with a fixed concentration of NPs (0.05 mg/ml). As shown in Fig. 2a, coumarin 6-LPN were easily internalized by HEI-OC1 cells after 1 h incubation. The uptake profile was in the form of a parabola (Fig. 2b, c). Furthermore, the cellular uptake increased with incubation time, reaching a peak at 3 h and then decreasing (p < 0.001).

### ATX-LPN attenuates CDDP-induced cytotoxicity

**Fig. 3:**
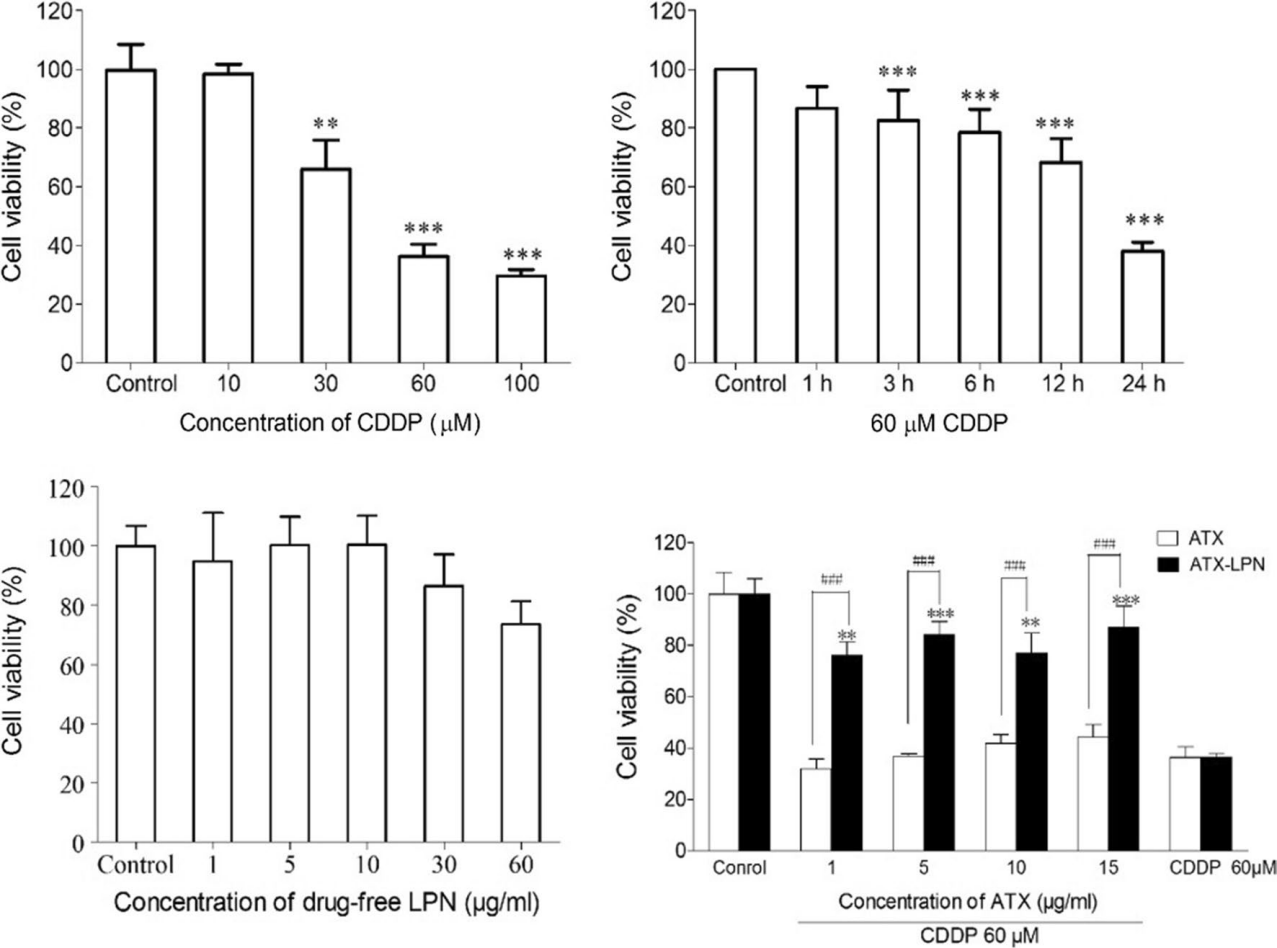
Cell viability of HEI-OC1 pretreated with ATX and ATX-LPN on CDDP-induced cell injury. **a** Cell viability of HEI-OC1 cells treated with a series of concentrations of CDDP (0, 10, 30, 60, 100 μM) for 24 h. **b** Cell viability of HEI-OC1 cells 60 μM CDDP at different incubation times (1, 3, 6, 12, and 24 h). **c** Cell viability of HEI-OC1 cells treated with drug free LPN.** d** Cell viability of HEI-OC1 cells pretreatment with ATX or ATX-LPN (1, 5, 10, 15 μg/ml) followed by 60 μM CDDP treatment for 24 h. **p < 0.01; ***p < 0.001 as compared with control. ^###^p < 0.001 as compared with ATX

To determine the optimal CDDP concentration for inducing HEI-OC1 cell damage, we used a series of concentrations (0, 10, 30, 60, and 100 μM) for 24 h. The cell viabilities were 100%, 98.445% ± 3.295%, 65.925% ± 10.039%, 36.234% ± 4.154%, and 29.584% ± 2.192% for each cisplatin concentration, respectively (Fig. 3a). In addition, we evaluated cell viability after 60 μM CDDP treatment for different times (1, 3, 6, 12, and 24 h). Only 24 h incubation exhibited 37.89% ± 3.18% (Fig. 3b). We chose the 60 μM CDDP treatment for 24 h as an appropriate condition for HEI-OC1 cell damage, on account of decrease in the number of viable cells to less than 50% that of the control. Hence, we used 60 μM CDDP treatment for 24 h for all experiments in this study.

We examined the cytoprotective effects of ATX/ATX-LPN at various concentrations (1, 5, 10, and 15 μg/ml) against CDDP injury. As shown in Fig. 3c, no cell ototoxicity of ATX-LPN was observed at the investigated ATX concentrations (1, 5, 10, and 15 μg/ml). The cell viability results showed that the percentages of viable cells in ATX-LPN + CDDP after treatment with experimental concentrations (1, 5, 10, and 15 μg/ml) were 76.03% ± 11.96%, 84.11% ± 11.43%, 76.83% ± 17.77%, and 87.13% ± 18.10%, which were significantly greater than those in the CDDP group (36.23% ± 4.15%) (Fig. 3d). However, the ATX + CDDP (1, 5, 10, and 15 μg/ml) groups showed no improvement in viable cells relative to the CDDP group. Together, these results suggested that ATX-LPN attenuated CDDP-induced cytotoxicity in HEI-OC1 under the experimental concentrations (1, 5, 10, and 15 μg/ml).

### ATX-LPN attenuates CDDP-induced apoptosis

**Fig. 4:**
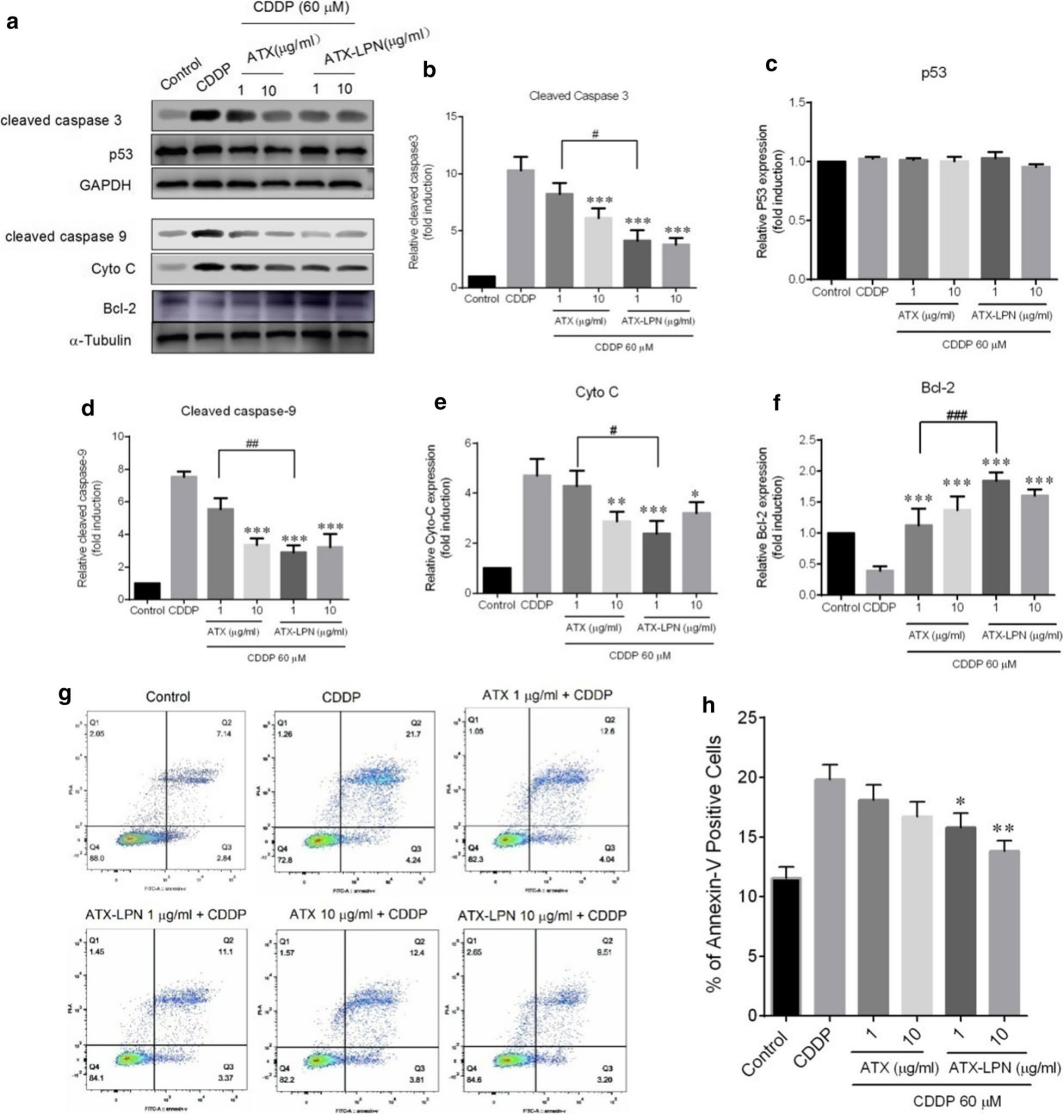
Cell apoptosis of HEI-OC1 pretreated with ATX, or ATX-LPN (1 and 10 μg/ml) for 4 h, followed by CDDP (60 μM, 24 h). **a** Western blots were performed with anti-cleaved-caspase 3, anti-cleaved caspase 9, anti-p53, anti-Cyto C, and anti-Bcl-2 antibodies. **b**–**f** Quantificantion of the western blots in **(a**). **g** Flow cytometry was used to measure the rate of apoptosis after different treatments. **h** Quantification of the data in (**g**). *p < 0.05, **p < 0.01, ***p < 0.001 as compared with CDDP. ^#^p < 0.05, ^##^p < 0.01, ^###^p < 0.001 as compared with ATX (1 μg/ml) + CDDP

As illustrated in Fig. 4a–f, the protein expression levels of pro-apoptotic proteins, cleaved-caspase 3/9, and cytochrome-c were significantly increased by CDDP (60 μM, 24 h) treatment but significantly suppressed by pretreatment with ATX (1 μg/ml) and ATX-LPN (1 or 10 μg/ml) separately. In addition, the expression level of the anti-apoptotic proteins Bcl-2 decreased with CDDP(60 μM, 24 h) treatment but was enhanced by pretreatment with ATX (1 or 10 μg/ml) and ATX-LPN (1 or 10 μg/ml).

Flow cytometry experiments were performed to evaluate cell apoptosis. The dead cells were labeled with PI, and the cells undergoing apoptosis were labeled with Annexin V. As shown in Fig. 4g, h, the ratio of apoptotic cells significantly increased after CDDP treatment (19.83% ± 2.44%), as compared with the control (11.54% ± 1.89%). Apoptotic cells (15.76 ± 2.45% and 13.80% ± 1.83%) significantly decreased after pre-treatment with ATX-LPN (1 or 10 μg/ml), ascompared with the CDDP group. However, pretreatment with ATX (1 or 10 μg/ml) did not result in fewer apoptotic cells than those in the CDDP group. Together, these results suggested that ATX-LPN significantly decreased CDDP-induced HEI-OC1 cell apoptosis at 1 and 10 μg/ml.

### ATX-LPN attenuates CDDP-induced oxidative damage and mitochondrial dysfunction

**Fig. 5:**
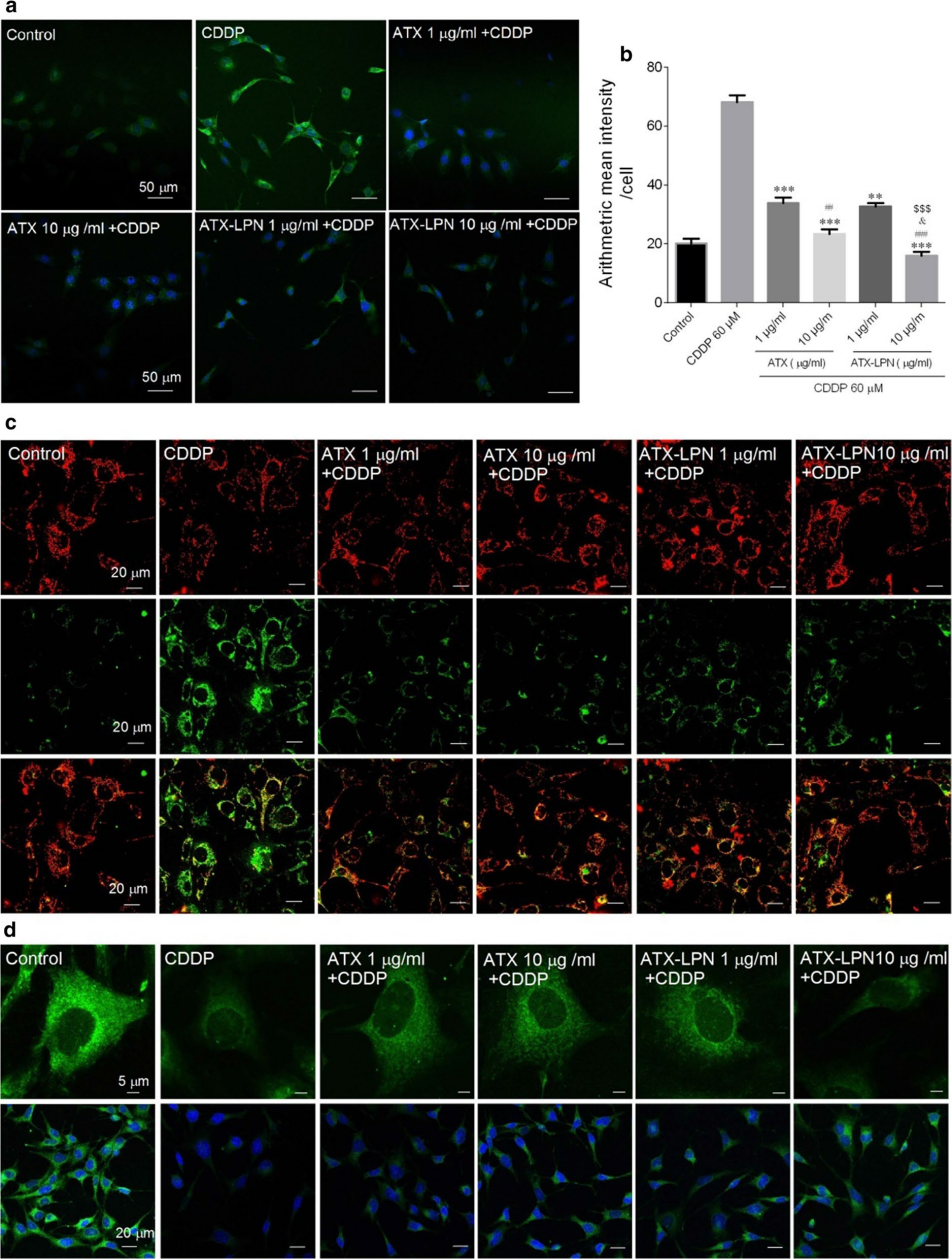
Detection of ROS level (DCFHDA) and Mitochondrial Dysfunction (JC-1 and MitoTracker Green) pretreated with ATX, or ATX-LPN (1 and 10 μg/ml) for 4 h, followed by CDDP (60 μM, 24 h). **a** Confocal images showing the intracellular ROS stained by DCFHDA (green fluorescence) in HEI-OC1 cells increased treated with 60 μM CDDP and was greatly inhibited by ATX or ATX-LPN. **b** Quantification of mean fluorescence intensity. ROS was significantly reduced after the application of ATX or ATX-LPN *p < 0.05, **p < 0.01, ***p < 0.001 as compared with CDDP. ^#^p < 0.05, ^##^p < 0.01, ^###^p < 0.001 as compared with ATX (1 μg/ml), ^&^p < 0.05 as compared with ATX (10 μg/ml), ^$$$^p < 0.001 as compared with ATX-LPN (1 μg/ml). **c** JC-1 staining has 2 forms: J -aggregate(red) at high MMPs and J-monomer(green) at low MMPs. **a** Change in fluorescence from red to green indicated a decrease of MMPs. **d** MitoTracker Green dying, representing the functional mitochondria in living cells

Previous studies have confirmed that CDDP triggers oxidative damage through inducing ROS accumulation. Therefore, we investigated the oxidative status in CDDP-treated HEI-OC1 cells. Intracellular ROS levels were monitored with the fluorescent probe DCFHDA [15]^.^ As shown in Fig. 5a, b, CDDP (60 μM, 24 h) treatment caused significant accumulation of ROS, as demonstrated by the green fluorescence in cells. However, pre-treatment with ATX or ATX-LPN (1 or 10 μg/ml) inhibited CDDP-induced generation of ROS. The statistical results further confirmed the anti-oxidative effects of ATX and ATX-LPN.

To elucidate the role of mitochondria in CDDP-induced apoptosis of HEI-OC1 cells, we detected MMP and morphology with JC-1 and MitoTracker Green, respectively. As shown in Fig. 5c, d, CDDP treatment (60 μM, 24 h) induced significant loss of MMP, as reflected by the fluorescence shift from red to green. ATX, or ATX-LPN (1 or 10 μg/ml) pre-treatment partially improved the MMP in HEI-OC1 cells. Moreover, CDDP treatment also caused clear mitochondrial fragmentation, as demonstrated by the mitochondrial morphological changes from protonema to punctiform. Interestingly, ATX or ATX-LPN (1 or 10 μg/ml) pre-treatment partially blocked CDDP-induced mitochondrial fragmentation.

### ATX-LPN attenuates the CDDP-induced activity of P-JNK

**Fig. 6:**
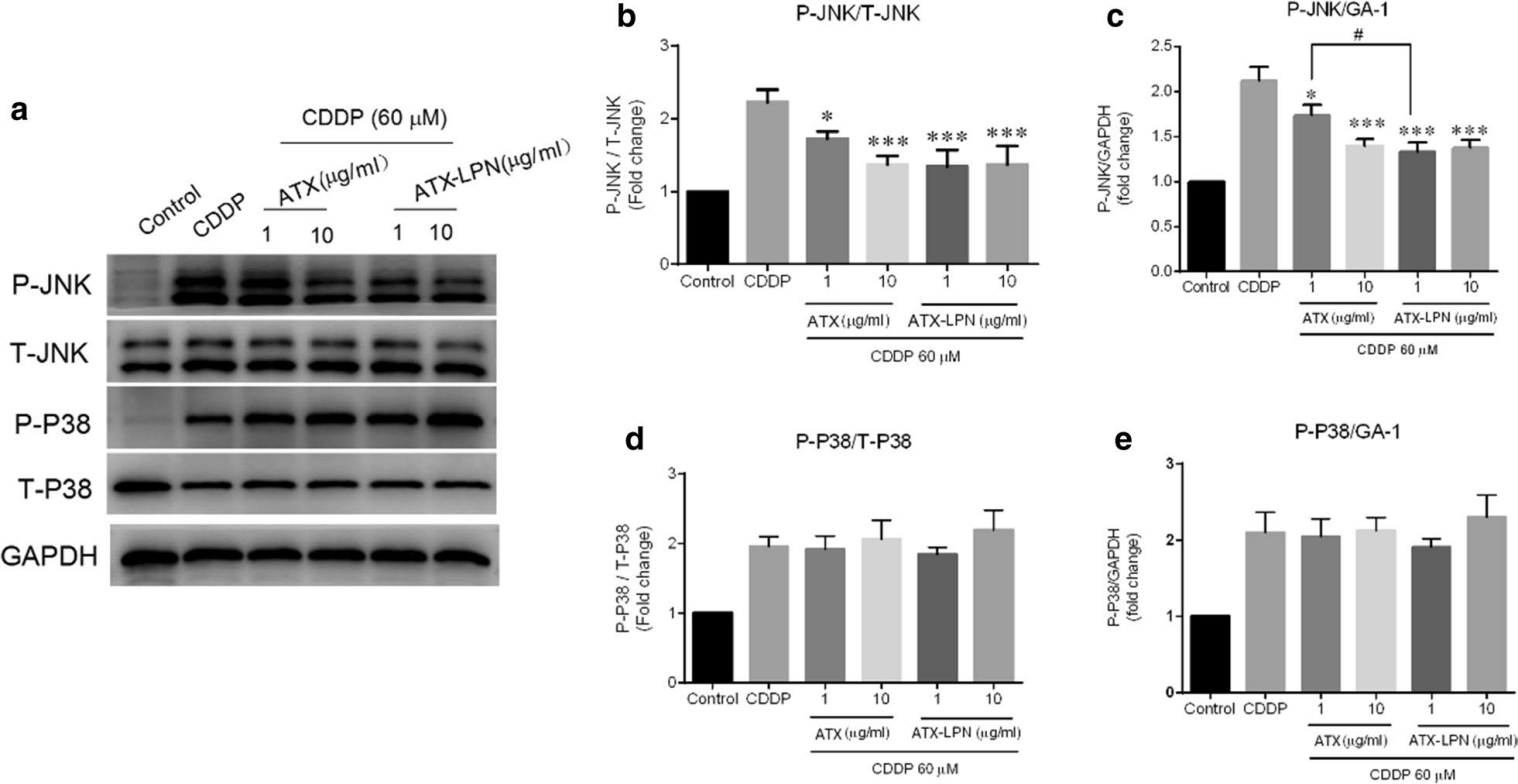
Western Blot analyses of the MAPK involvement. **a** HEI-OC1 cells were treated with CDDP and ATX/ATX-LPN as above. The expression of JNK and p38 were evaluated by WB. **b**–**e** Quantification of the light intensity of the protein expression from western blot results above. ATX or ATX-LPN administration on CDDP-treated HEI-OC1 lead to a decreasing ratio of P-JNK/T-JNK (**b**) and P-JNK/GAPDH (**c**), with no change in that of P-P38/T-P38 (**d**) and P-P38/GAPDH (**e**). *p < 0.05, **p < 0.01, ***p < 0.001 as compared with CDDP. ^#^p < 0.05 as compared with ATX (1 μg/ml),

As shown in Fig. 6, CDDP (60 μM, 24 h) activated JNK and p38, as characterized by an increase in the degree of phosphorylation in total JNK and p38. However, the anti-inflammatory effects of 1 or 10 μg/ml ATX or ATX-LPN on CDDP-treated HEI-OC1 were quite different, as reflected by a significant decrease in the ratio of P-JNK/T-JNK and P-JNK/GAPDH, whereas nearly no change was observed in the P-P38/T-P38 and P-P38/GAPDH ratios. More specifically, 1 μg/ml ATX-LPN decreased the activity of JNK more effectively than 1 μg/ml ATX (p < 0.05).

## ATX-LPN protective effect against damage to zebrafish hair cells

**Fig. 7:**
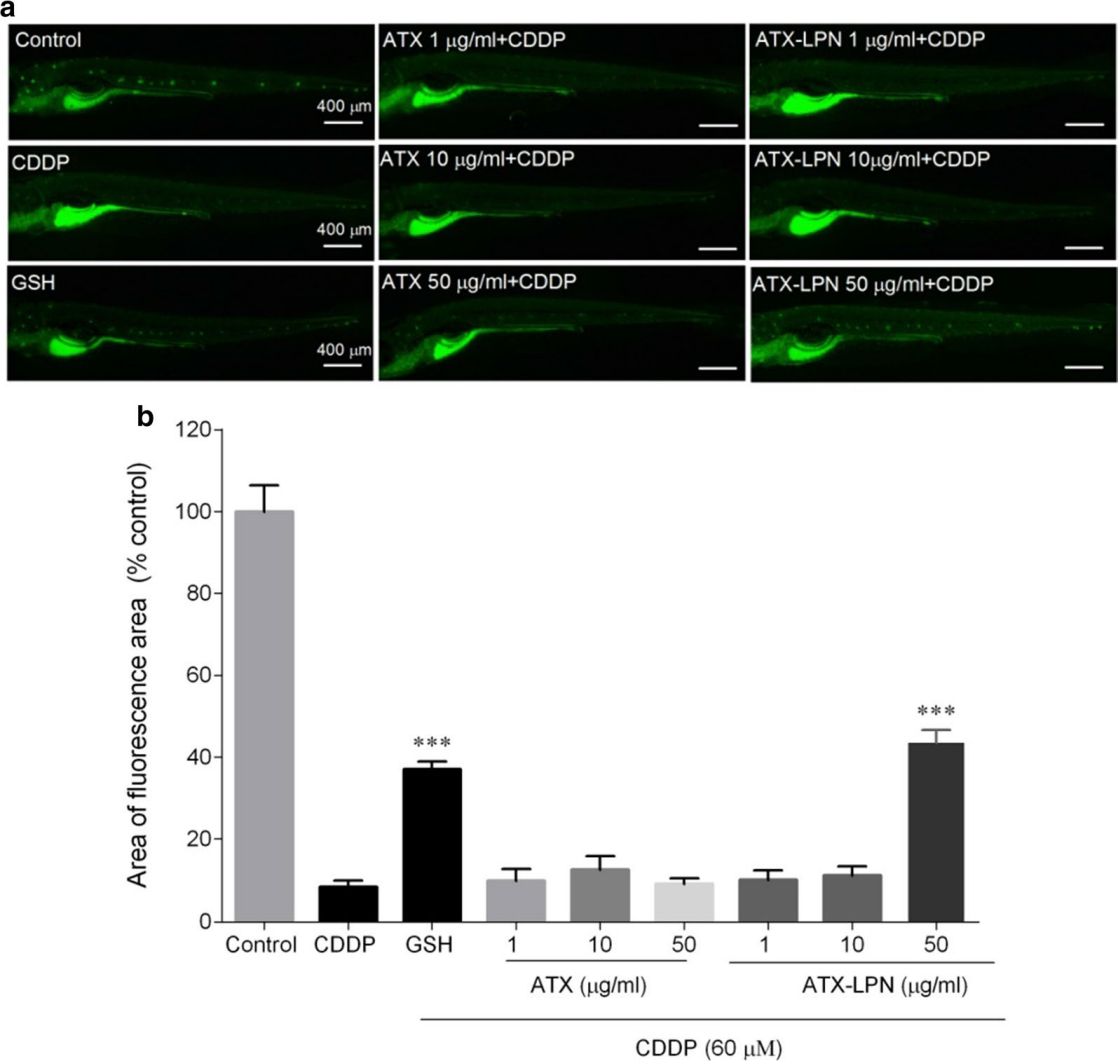
Evaluation of neuromast hair cell damage by DASEPI in zebrafish **a** Neuromasts hair cells of zebrafish, stained by DASEPI (green), demonstrated the damage of CDDP and protection of ATX/ATX-LPN. **b** Calculation of the protective percentage of ATX/ATX-LPN. ***p < 0.001 as compared with CDDP

We evaluated the neuronal ATX hair cell damage by examining the fluorescence intensity of DASEPI and calculating the protective effect with the formula above (Fig. 7). Both ATX and ATX-LPN at concentrations of 1 and 10 µg/ml did not decrease the damage to hair cells induced by CDDP. Only at the concentration of 50 μg/ml did ATX-LPN had an clear protective effect (32%) against the damage to zebrafish hair cells, to a level greater than that of the GSH (154 µg/ml) positive control (31%).

### ATX-LPN rescue OHCs damage in cultured organ of Corti

**Fig. 8:**
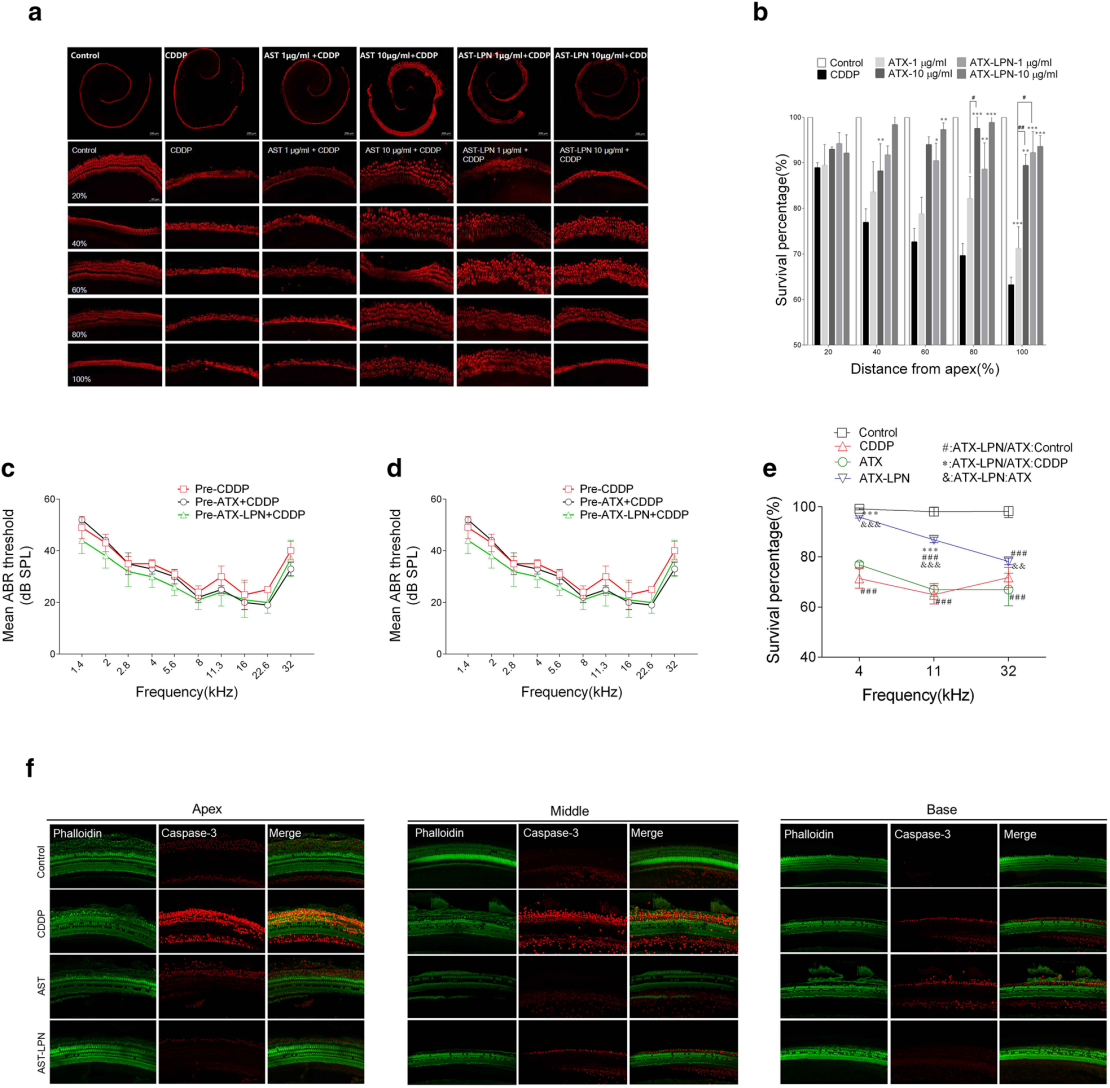
ATX-LPN partially rescue CDDP-induced hearing loss. **a** In vitro culture of Organ of Corti. CDDP induced a significant loss of hair cells (Myosin VII, red), especially in the high-frequency areas. **b** Survival outer hair cell numbers in 1 mm length at different locations from apical end of each group. *p < 0.05, **p < 0.01, ***p < 0.001 as compared with CDDP, ^#^p < 0.05 as compared with ATX + CDDP. **c**–**e** Mean ABR threshold of mice treated with cisplatin, ATX 1 mg/ml, 5 μl. ATX-LPN + CDDP. *p < 0.05, **p < 0.01, ***p < 0.001 as compared with CDDP, ^#^p < 0.05 as compared with ATX + CDDP. **f** Immunohistochemistry of cochlea in apex/middle/basal area of BM. The pretreatment of protective drugs (AST/AST-LPN) effectively reduced the expression of caspase-3 (red fluorescence) and rescued more OHCs (green fluorescence)

As shown in Fig. 8, CDDP induced a significant loss of hair cells and showed a greater disruption in the high-frequency area. Morphologically, the arrangement of OHCs was irregular, and vacancies in OHCs occurred (Fig. 8a). The treatment with ATX/ATX-LPN attenuated the damage to OHCs induced by CDDP, and a significant difference was observed in the 1 and 10 μg/ml ATX-LPN groups in the middle and basal basilar membrane (60%, 80%, and 100% from the apex) (Fig. 8b); 10 μg/ml ATX-LPN rescued the greatest amount of OHCs (30.33% ± 0.71%, 29.21% ± 1.80%, and 24.63% ± 1.91%). In addition, 10 μg/ml ATX rescued a significant amount of OHCs in the locations of 40%, 80%, 100% from the apex (11.28% ± 3.04%, 27.88% ± 1.43%, and 26.16% ± 4.14%), as did 1 μg/ml ATX at the basal part (100% from the apex) of the basilar membrane, 8.01% ± 5.24%. (*p < 0.05; **p < 0.01; ***p < 0.001). A higher concentration of ATX had a better effect in rescuing the OHCs at the basal part of the BM (80% and 100% from the apex), and no influence of concentration was observed for ATX-LPN. At the same concentrations, ATX and ATX-LPN showed no significant difference in decreasing the loss of OHCs, except at 1 μg/ml at 100% from the apex.

### ATX-LPN partially restored CDDP-induced hearing loss in ABR tests

Conventional ABR recording was used to monitor auditory function after CDDP treatment. As shown in Fig. 8c–e, the ABR tests indicated that ATX-LPN partially protected against CDDP-induced hearing loss on day 3 after RWM administration. Compared with CDDP, ATX-LPN + CDDP exhibited a significantly enhanced protective effect at 2 kHz (p < 0.05), 2.8 kHz (p < 0.001), 4 kHz (p < 0.001), 5.6 kHz (p < 0.001), 8 kHz (p < 0.001), 11.3 kHz (p < 0.001), 16 kHz(p < 0.001), and 22.6 kHz(p < 0.001) frequencies. However, the ATX + CDDP group did not show a statistically significant difference in the mean ABR threshold at all detected frequencies. Furthermore, ATX-LPN + CDDP showed a greater preventive effect than ATX + CDDP at 2 kHz (p < 0.05), 2.8 kHz (p < 0.001), 4 kHz (p < 0.001), 5.6 kHz (p < 0.001), 8 kHz (p < 0.001), 11.3 kHz (p < 0.001), 16 kHz (p < 0.001), and 22.6 kHz (p < 0.01) frequencies.

Hair cells were stained with FITC-conjugated phalloidin. Caspase-3, a downstream effector caspase that executes apoptosis, was also observed to be mainly distributed in the supporting cells in control guinea pig cochleae. Morphologically, CDDP caused irregular formation and several vacancies in the first row of OHCs in cochlea, whereas caspase-3 expression was greatly enhanced in the second and third rows. Pretreatment with protective drugs (ATX/ATX-LPN) effectively weakened the fluorescence of caspase-3 and rescued more OHCs. Specifically, ATX decreased the expression of caspase-3 but did not inhibit the hair cell loss, whereas ATX-LPN showed a stronger effect on attenuating the expression of caspase-3 as well as rescuing OHCs (Fig. 8f).

## Discussion

CDDP, an antineoplastic agent, is indispensable in clinic settings; however, ototoxicity usually restricts its wide use. Substantial effort has been made to explore new agents to alleviate or prevent CDDP-induced ototoxicity. Although no agents have been approved by the FDA to date, numerous agents have been successful in preclinical work, and several have shown protection in the clinic. Among them, sodium thiosulfate is the only agent exhibiting protective effects against CDDP-induced ototoxicity in clinical settings [2]. The mechanism is not fully understood, but antioxidation and anti-inflammation are assumed to be the major causes. In the present study, we established LPN for loading ATX to successfully facilitate penetration through the RWM and maintain ATX concentrations in the inner ear perilymph for 24 h after a single injection. ATX-LPN have favorable biocompatibility and strongly affect CDDP-induced generation of ROS in HEI-OC1 cells. JC-1 and MitoTracker Green staining suggested that ATX-LPN successfully reversed the decrease in MMP induced by CDDP in vitro, as well as rescuing cells in early stages of apoptosis, as demonstrated by FACS. Moreover, ATX-LPN successfully attenuated OHCs losses in animal models (zebrafish and guinea pigs) in vivo.

ATX, with its superior anti-oxidant and anti-inflammatory properties, exhibits protective effects in cardiovascular health and disease, ischemia and reperfusion injury, cognitive function with age, and neurodegeneration [16]. However, its use in the food, feed, and pharmaceutical fields is limited, owing to low bioavailability, poor stability during thermochemical treatments, susceptibility to oxidation, and poor organoleptic characteristics. Nanoparticle-based drug delivery systems have been used to encapsulate ATX to avoid the drawbacks mentioned above. Recently, many drug delivery systems and preparation methods have been explored to improve stability and water-solubility, such as DNA/chitosan nanoparticles [17], polyrrole nanoparticles [18], gold nanoparticles [19], nanoliposomes [20], nanopowders, and nanodispersions [20]. In the present study, we fabricated ATX-LPN by using an emulsion and evaporation technique. The LPN are composed of a phospholipid shell and polymer core, thereby providing a core–shell structure with the advantages of both liposomes and polymer nanoparticles [21, 22]. The solid polymer core endows the LPN with mechanical stability, shape control, biodegradability, a narrow particle-size distribution, and a large surface area, and serves as a structural framework [23]. In addition, the lipid shell of the LPN, with properties similar to those of cell membranes, easily combines with a variety of bioactive molecules [24]. Hence, LPN have attracted attention as a new type of drug carrier in recent years. We optimized the preparation method by comparing the method of nanoprecipitation and emulsion solvent evaporation, the latter of which improved encapsulation efficiency and drug loading, thus resulting in a higher solubility of ATX in dichloromethan (Fig. 1).

Although systemic administration is an ideal drug delivery method for inner ear therapy, ATX and even ATX-LPN could not pass the BBB and BLB after IP injection with as high as 200 µg/ml (Fig. 1f). Local drug delivery, including intratympanic and intracochlear delivery, provides an alternative pathway. The lipid shell of the LPN improved the ATX water solubility and altered its size, and ATX-LPN easily penetrated RWM and entered the endolymph in the inner ear. ATX-LPN exhibited a long-term release profile in vitro (15 d) thus suggesting long retention in vivo (24 h).

In the inner ear, many antioxidants, such as rosmarinic acid [25], alpha-lipoic acid [26], and Mitoquinone [27] have been applied to improve hearing loss after exposure to various damage, such as noise or ototoxic drugs. ATX, which has a stronger biological activity than other antioxidants because of its link with the cell membrane from the inside to the outside [28], has been shown to protect against CDDP-induced nephrotoxicity in rats [29] and neomycin-induced ototoxicity in zebrafish hair cells [30]. However, to our knowledge, the effect of ATX on CDDP-induced ototoxicity has not previously been studied. In the present study, we provide evidence that ATX-LPN successfully attenuated OHC losses both in cultured organ of Corti and in animal models (zebrafish and guinea pigs).

The mechanism of CDDP-induced ototoxicity is complex and remains unclear. Among possible hypotheses, generation of excessive ROS, such as superoxide radicals, hydrogen peroxide, and hydroxyl radicals, have been implicated as major contributors [31]. Either increased toxic lipid peroxides or depletion of cochlear antioxidant enzyme activities can lead to a cascade resulting in apoptosis of hair cells [2]. Many studies have shown favorable effects of ATX on the regulation of oxidative stress, in accordance with our finding that ATX-LPN significantly attenuated the production of ROS induced by CDDP. ATX inhibits mitochondrial production of intracellular ROS via inducing expression of antioxidants (e.g., superoxide dismutase) [32], scavenging oxidative stress products (e.g., 4-hydroxy-2-nonenal, 4-HNE) [33, 34], and retaining the mitochondrial redox state and functional integrity [35]. Intriguingly, Niu et al. have demonstrated that ATX generates trace amounts of ROS, thus activating the cellular antioxidant defense system via the ERK-Nrf-2/HO-1 pathway in HUVECs [36].

The present study confirmed the CDDP ototoxicity on hair cells through induction of apoptosis. Apoptosis occurs through two main pathways: extrinsic and intrinsic. The former is triggered by FAS, and the latter originates from a release of cytochrome-c. Both pathways are followed by activation of a cascade of caspases [37]. In the present study, CDDP led to apoptosis in both intrinsic and extrinsic pathways, as evidenced by the decreased levels of cleaved caspase 3/9 and cytochrome-c, and the increased expression of BCL-2, which represses apoptosis by blocking the release of cytochrome-c, in accordance with findings from previous studies [38]. Encouragingly, ATX/ATX-LPN attenuated the levels of the apoptotic proteins mentioned above in the intrinsic and extrinsic pathways, and consequently inhibited apoptosis. Meanwhile, levels of one of the most important proteins that regulates apoptotic pathways, p53 (a transcription factor regulating downstream genes important in cell cycle arrest, DNA repair, and apoptosis), remained constant after the administration of CDDP. Conversely, p53 has been demonstrated to be a major determinant of CDDP ototoxicity, and genetic or pharmacological ablation of p53 substantially attenuates cochlear cell apoptosis [39]. Another study has demonstrated that ATX treatment enhances expression of P53 [40]. Thus, the level of P53 appears to depend on the balance between its pro-apoptotic and anti-apoptotic functions, which is affected by phosphorylation status, subcellular localization, or protein interactions.

Phosphorylation of STAT1, a transcription factor involved in inflammation and apoptosis, can be achieved by serine kinases such as ERK 1/2, p38, and JNK [41]. These kinases are stimulated by ROS, which is increased after the use of CDDP, as evidenced above. In the present study, the activation of JNK induced by CDDP was upregulated and reduced after the administration of ATX/ATX-LPN, while P38 remained unchanged. However, another study has demonstrated that inhibition of the JNK signaling pathway does not prevent the CDDP-initiated activation of participants in apoptosis despite a significant increase in activated JNK [38]. Similarly, a previous study [42] has demonstrated that after IL-1β stimulation, ATX decreased the phosphorylation of two mitogen-activated protein kinases, p38 and ERK1/2, in chondrocytes. Another study has found that ATX induces thephosphorylation of ERK kinase in HUVECs [36]. Together, these studies indicate that mitogen-activated protein kinase signaling pathways are involved in the protective process of ATX in CDDP-induced ototoxicity. The discrepancymay be attributed to different cells and different treatments.

The protective effect of ATX-LPN in terms of morphology was verified by rescuing OHCs in cultured cochlear explants. We then evaluated auditory function and found that hearing was restored by 20–30 dB SPL at a wide range of frequencies after ATX/ATX-LPN administration. Although greater CDDP-induced damage exists in the high-frequency area in both animals and humans [43], a better restoration of hearing was observed. ATX-LPN showed stronger protection of OHCs at the basal part of the BM in both cultured organ of Corti and guinea pig cochleae. These effects cannot be explained by a higher accumulation of drug at the base of the BM through RWM penetration, and the intrinsic characterization of the basilar membrane may play an important role.

## Conclusion

In summary, in the present study, we developed a nanoparticle delivery system for loading ATX to rescue the ototoxicity induced by CDDP. The synthesized ATX-LPN were uniformly spherical in shape and showed favorable chemical stability and biocompatibility. Our results clearly demonstrated that ATX-LPN decreased ROS generation and MMP collapse, inhibited CDDP-induced hair cell apoptosis, and eventually attenuated hair cell loss both in vitro and in vivo. Although the detailed mechanisms must be further investigated, the results suggest that ATX-LPN may be a potential drug for alleviating CDDP-induced hearing loss.

## Methods

### Materials, cell culture and animals

ATX, DMSO, dichloromethane, and cholate solution were purchased from Sigma-Aldrich (USA). CDDP was purchased from Aladdin (Shanghai, China). Methoxy (polyethylene glycol) (mPEG-PLA, MW 50 kDa) was purchased from Daigang Biomaterial Co, Ltd (Ji’nan, China). DMPC was purchased from Avanti Polar Lipids, Inc (USA). DAPI was purchased from Invitrogen (Carlsbad, CA, USA).

The House Ear Instituteorgan of Corti 1 (HEI-OC1) cell line (generated at the House Ear Institute, Los Angeles, CA, USA) was cultured in DMEM (Gibco) supplemented with 10% fetal bovine serum (Gibco, Waltham, MA, USA) free of antibiotics at 33 °C under 10% CO_2_.

Healthy male guinea pigs (4–6 weeks of age, weighing 220–250 g) without otitis media were purchased from the Shanghai Laboratory Animal Center (Shanghai, People’s Republic of China). All animal procedures were carried out in accordance with the Ethical Committee of Shanghai Jiao Tong University School of Medicine (Shanghai, China). Animal experiments complied with the 3R principles (reduction, replacement, and refinement).

### Preparation of ATX-loaded lipid-polymer nanoparticles (ATX-LPN)

ATX-LPN were fabricated with a single emulsion solvent evaporation method. Briefly, mPEG-PLA and ATX (6:1, w/w) were mixed and completely dissolved in 1 ml of dichloromethane solution as the oil phase. DMPC was then dissolved in 3 ml of sodium cholate solution (1%, w/v) as the aqueous phase. The oil phase and aqueous phase were mixed well and sonicated at 390 W for 30 s (Scientz Biotechnology Co, Ltd, Ningbo City, People’s Republic of China). The resulting emulsion was further diluted in 36 ml of 0.5% sodium cholate solution, and the oil phase was removed by evaporation. The polymer core formed, and the lipid self-assembled around it. Finally, LPN were acquired by centrifugation (11,000×*g*, 30 min) and washed twice to remove excess emulsifier. ATX-LPN were frozen and stored at − 20 °C for further study.

### Morphology and characterization of ATX-LPN

The particle size, zeta potential and polydispersity index (PDI) were determined with a Zetasizer Nano-ZS instrument (Particle Sizing Systems, Santa Barbara, CA, USA). Triplicate measurements for each sample were carried out. ATX-LPN were negatively stained with sodium phosphotungstate solution and scanned with a transmission electron microscope (Hitachi, Tokyo, Japan) to examine the morphology. The encapsulation efficiency (EE%) and drug loading (DL%) of the ATX-LPN were examined with LC–MS/MS, as described in a previous study [44]. The methode in detail was described as the the following " LC–MS/MS method for ATX" section.

### In vitro and in vivo release profiles

In vitro release profile analysis was performed in artificial perilymph (pH7.4) at 37 °C in a water bath. ATX-LPN (0.2 mg/ml) suspension was placed in a centrifuge tube and diluted in artificial perilymph to 1 ml. At specific time points (0.5, 1, 6, 12 h, 1 d, 2 d, 5 d, 10 d, and 14 d), the tube was removed and centrifuged, and 900 μl supernatant was acquired and stored at − 20 °C for ATX detection. The tube was refilled with 900 μl of fresh artificial perilymph for the next release measurement.

The in vivo release profile was determined in guinea pigs divided into three groups: group I, intraperitoneal injection (IP) administration of 200 μg ATX; group II, RWM administration of 6 μg ATX; and group III, RWM administration of 6 μg ATX-LPN. The guinea pigs were decapitated at different time points, and the left cochlea was removed. The RWM was punctured with a glass electrode, approximately 7 μl perilymphatic fluid was extracted with a 1 ml syringe, and the EP tube containing external lymphatic fluid was stored at − 80 °C. The obtained buffer was subjected to LC–MS/MS determine the ATX concentration, and the minimum detection limit was 1 ng/ml.

### LC–MS/MS method for ATX

The LC system comprised a Shimadzu (Shimadzu Co., Japan) liquid chromatography equipped with a binary pump (LC-30AD), an autosampler (SIL-30AC), a column oven (CTO-20A), a system controller (CBM-20A) and a degasser (DGU-20A). Mass spectrometric analysis was performed using an AB SCIEX API6500 triple-quadrupole (Ontario, Canada) instrument with an ESI interface. The data acquisition and control system were created by using Analyst 1.6.2 software from AB SCIEX (Ontario, Canada). Chromatographic separation on a Waters X-Bridge BEH C18 (2.1 × 50 mm, 2.5 µm), mobile phase A is water (containing 1% formic acid), mobile phase B is ACN (containing 1% formic acid), The column was eluted at a flow rate of 0.6 ml/min in a gradient program consisting of 2% phase B (0–0.4 min), from 2 to 65% B (0.4–0.7 min), from 65 to 90% B (0.7–1.30 min), 90% B (1.30–1.90 min), from 90 to 2% B (1.90–1.91 min), 2% B (1.91–2.50 min). For Astaxanthin, the retention time for the analyte and IS (Diclofenac) were 1.82 min and 1.30 min respectively, injection volume is 5 µl.

The precursor product ion pair was m/z 597.3 → 147.1 for Astaxanthin, m/z 296.2 → 214.2 for Diclofenac.

### Standard curve preparation

Stock solution of Astaxanthin were prepared at 1 mg/mL in DMSO. The stock solution was diluted with MeOH to preparing serial working solution (200, 400, 600, 1000, 2000, 6000, 20,000 ng/ml), an aliquote of 10 µl working solution was spiked into 190 µl guinea pig plasma: PBS 1:9 (V/V) to obtain calibration standard curve (10, 20, 30, 50, 100, 300, 1000 ng/ml).

### Sample preparation

An aliquot of 3 µl sample was diluted with 12 µl guinea pig plasma: PBS 1: 9 (V/V). Then 75 µl IS (Diclofenac, 100 ng/ml in ACN) was added for protein precipitation. The mixture was vortexed for 10 min at 750 rpm and centrifuged at 6000 rpm for 10 min. An aliquot of 5 µl supernatant was injected for LC–MS/MS analysis.

### Cellular uptake by HEI-OC1 cells

To trace LPN uptake by HEI-OC1 cells, we encapsulated coumarin 6 in LPN and used DAPI to label nuclei. Briefly, HEI-OC1 cells were cultured on VWR Micro Cover Glasses in 24-well plates at a density of 5 × 10^4^ cells/well. When the cells reached approximately 80% confluence, the medium was replaced with coumarin 6-labeled LPN and incubated for 1, 3, 6, and 12 h. After the coumarin 6-LPN were removed, and the wells were washed twice with PBS, the cells were fixed with 4% glutaraldehyde for 20 min, and the cell nuclei were stained with DAPI for 30 s. The cells were then observed under an LSM-510 CLSM (Carl Zeiss AG, Oberkochen, Germany) with a fluorescein isothiocyanate (FITC) filter (excitation, 488 nm; emission, 520 nm), and flow cytometry (BD LSR Fortessa, America) was performed.

### Cell viability

The in vitro cytotoxicity of nanoparticles was assessed with CCK-8 assays. HEI-OC1 cells were seeded at a density of 5 × 10^3^cells/well in a 96-well plate and allowed to attach overnight. Then they were divided into the following three groups of exposures: ATX or ATX-LPN at various concentrations (1, 5, 10, and 15 μg/ml); CDDP (10, 30, 60, and 100 μM); and co-treatment with CDDP (60 μM) and ATX/ATX-LPN (1, 5, 10, and 15 μg/ml). After a 24 h incubation, 10 μl CCK-8 reagent (Dojindo Molecular Technologies, Japan) was added to each well and reacted for 2 h at 37 °C in 5% CO_2_. The absorbance was measured at 450 nm with a plate reader. The percentage of cell viability was calculated by comparing cells treated with different formulations to the corresponding control cells.

### ROS evaluation

The fluorescent probe 2, 7-dichlorofluorescein diacetate (DCFH-DA) was used to detect intracellular ROS production. HEI-OC1 cells, seeded at a density of 5 × 10^5^ and cultured overnight, were treated with 60 μM CDDP alone or co-treated with 1, or 10 μg/ml ATX/ATX-LPN for 24 h, then incubated with 10 μM DCFH-DA (Abcam, MA, USA) in serum-free medium at 37 °C for 30 min in the dark. Quantification of the fluorescence intensity (488 nm excitation/525 nm emission) was performed with a Zeiss confocal laser scanning microscope.

### Detection of mitochondrial membrane potential

JC-1 (Thermo Fisher Scientific, MA, USA) and MitoTracker Green (Thermo Fisher Scientific, MA, USA) are widely used fluorescent dyes for monitoring mitochondrial membrane protentional (MMP). HEI-OC1 cells were cultured and treated with 60 μM CDDP alone or co-treated with 1 or 10 μg/ml ATX/ATX-LPN for 24 h, then stained with 10 μM JC-1 and 1 μM MitoTracker Green for 20 min and 45 min, respectively. JC-1 exhibits double fluorescence staining, either as red fluorescent J-aggregates (530 nm excitation/590 nm emission) at high potentials or as green fluorescent J-monomers (490 nm excitation/530 nm emission) at low potentials; a fluorescence change from red to green represents a decrease in MMP. MitoTracker Green, another fluorescent stain for mitochondria, labelsfunctioning mitochondria in living cells and exhibits green fluorescence (490 nm excitation/516 nm emission). Images were acquired with a Zeiss confocal laser scanning microscope.

### Apoptosis assays

Flow cytometry was carried out for quantitative evaluation of apoptosis. HEI-OC1 cells were seeded in six-well culture plates at a density of 5 × 10^5^ per well for 24 h before the assay. After incubation with 60 μM CDDP alone or co-treatment with 1 or 10 μg/ml ATX/ATX-LPN for 24 h, treated cells were trypsinized, collected, washed with PBS, and resuspended. The cells were then incubated with FITC-conjugated Annexin V (BD biosciences) and PI (BD biosciences) for 15 min at room temperature in the dark. Annexin V-FITC/PI positive cells were analyzed by fluorescence-activated cell sorting (FACS) (Becton Dickinson).

### Western blotting

To determine levels of proteins in the apoptosis pathway, we treated HEI-OC1 cells with 60 μM CDDP and 1 or 10 μg/ml ATX/ATX-LPN as described above, and then harvested the cells for western blot analyses. The protein extracts were subjected to 10% SDS-PAGE and electrotransferred to PVDF membranes. The membranes were then blocked for 1 h in quick-blocker at room temperature and incubated overnight in a cold chamber (4 °C) with specific primary antibodies. After incubation, the membranes were washed with TBS and then incubated with HRP conjugated secondary antibody for 1 h at room temperature, washed repeatedly, and visualized with an enhanced chemiluminescence kit (Thermo Fisher Scientific). GAPDH and α-tubulin served as internal standards. The relative intensity was quantified by Quantity One. The following primary antibodies were used: anti-cleaved-caspase 3, anti-cleaved-caspase 9, anti-p53, anti-BCL-2, anti-P-P38, anti-T-P38 and anti-cytochrome 3 (Cell Signaling Technology, CST), anti-P-JNK (Abcam), and anti-T-JNK (Abcam).

### Evaluation of hair cell damage on zebrafish

This experiment was carried out by Hunter Biotechnology,Inc (Hangzhou, China). Briefly, zebrafish larvae (5 days post-fertilization, n = 30 per concentration) were pretreated with various concentrations (1, 10 and 50 µg/ml) of ATX (1, 10 and 50 µg/ml), ATX-LPN (1, 10 and 50 µg/mL) or GSH (154 μg/mL) for 4 h, then exposed to CDDP (60 μM) for 24 h. After being washed three times, larvae were stained with 2-[4-(dimethylamino)styryl]-1-ethylpyridinium iodide) (Sigma) to evaluate the hair cell damage of neuromasts. Then ten zebrafish from each experimental group were randomly selected and photographed under a fluorescence microscope, and the images were analyzed in Image-J. The fluorescence intensity (S) of neuromast hair cells in the zebrafish body was calculated. The below formula was used to evaluate the damage to hair cells: Protective effect on hair cell damage (%) = [S(treatment) − S(CDDP)]/[S(control) − S(CDDP)] x 100%.

### Administration of ATX-LPN on RWM

RMW administration was described in our previous study [45], Briefly, guinea pigs were anesthetized with an intraperitoneal (IP) injection of 1% pentobarbital sodium (35 mg/kg). During the procedure, the animals were placed on a heating pad (38 °C). A subcutaneous injection of 1% lidocaine was performed to reduce pain. A postauricular incision was made and the muscle was dissociated via blunt dissection and retracted until exposure of the auditory bulla. A hole of 2–3 mm in diameter was drilled on the bulla to provide direct visualization of the round window niche. A ATX or ATX-LPN solution (6 μl) was applied onto the RWM using a microsyringe. The guinea pigs were fixed at this position for 30 min. The bulla opening was sealed with dental cement and the incision was closed with sutures.

### Evaluation of hearing loss and hair cell damage in guinea pigs

Auditory brainstem responses (ABR) were measured in anesthetized animals before and 3 days after the drug administration. Guinea pigs were placed on a heating pad maintained at 37 °C in a sound-proof booth. Pure tone stimuli of 1.4, 2, 2.8, 4, 5.6, 8, 11.3, 16, 22.6, or 32 kHz between 0 and 90 dB SPL in 5 dB steps were delivered through an open-field microphone. Subcutaneous electrodes collecting signals were inserted at the pinna (recording electrode), vertex (reference electrode), and rump (ground electrode). The acoustic signals were generated with Tucker-Davis Technologies (Alachua, FL, USA) hardware. Thresholds were defined as the lowest stimulus level at which a response was observed.

Guinea pig cochleae from the three groups were perfused with 4% paraformaldehyde for 2 h for fixation and decalcified in 0.12 mM EDTA at room temperature for 1 week. The cochleae were then microdissected into the apex, middle and basal turns and subjected to immunohistostaining of FITC-conjugated phalloidin (green) and caspase-3 (red), indicating stereocilia and apoptosis. Specimens were mounted on slides with anti-fade mounting medium and imaged with a Zeiss confocal microscope. The percentage of survival OHCs at the basal, middle and apex turns was determined for areas located at 4, 11, and 32 kHz.

### Statistical analysis

All statistical analyses were performed in GraphPad Prism. Variables are expressed as means ± standard error of mean (SEM). Student's t-test and two-way ANOVA with Bonferroni correction were used for statistical analysis. Values of P < 0.05 were considered significant.

## Data Availability

Date and material are availability for any research.

## Abbreviations

FDA: Food and drug administration
ROS: Reactive oxygen species
ATX: Astaxanthin
HEI-OC1: House ear instituteorgan of corti 1
RWM: Round window membrane
LPN: LIpid-polymer nanoparticles
DCFHDA: 2,7-Dichlorodi-hydrofluorescein diacetate
FACS: Fluorescence activating cell sorter
CDDP: Cisplatin
PDI: Polydispersity index
DMPC: 1,2-Dimyristoyl-sn-glycero-3-phosphocholine
mPEG-PLA: Methoxy (polyethylene glycol)
