# Correction to: Astaxanthin-loaded polymer-lipid hybrid nanoparticles (ATX-LPN): assessment of potential otoprotective effects

**DOI:** 10.1186/s12951-020-00627-0

**Published:** 2020-05-19

**Authors:** Jiayi Gu, Yuming Chen, Ling Tong, Xueling Wang, Dehong Yu, Hao Wu

**Affiliations:** 1 Department of Otolaryngology-Head and Neck Surgery, Shanghai Ninth People’s Hospital, Shanghai Jiao Tong University, School of Medicine, Shanghai, 200011 China; 2 Ear Institute, Shanghai Jiao Tong University, School of Medicine, Shanghai, 200011 China; 3 Shanghai Key Laboratory of Translational Medicine On Ear and Nose Diseases (14DZ2260300), Shanghai, 200011 China

## Correction to: J Nanobiotechnol (2020) 18:53 10.1186/s12951-020-00600-x

Following publication of the original article [1], the authors identified errors in Fig. 8d and f. The corrected Fig. 8 and the corrected figure caption are given below.

**Fig. 8:**
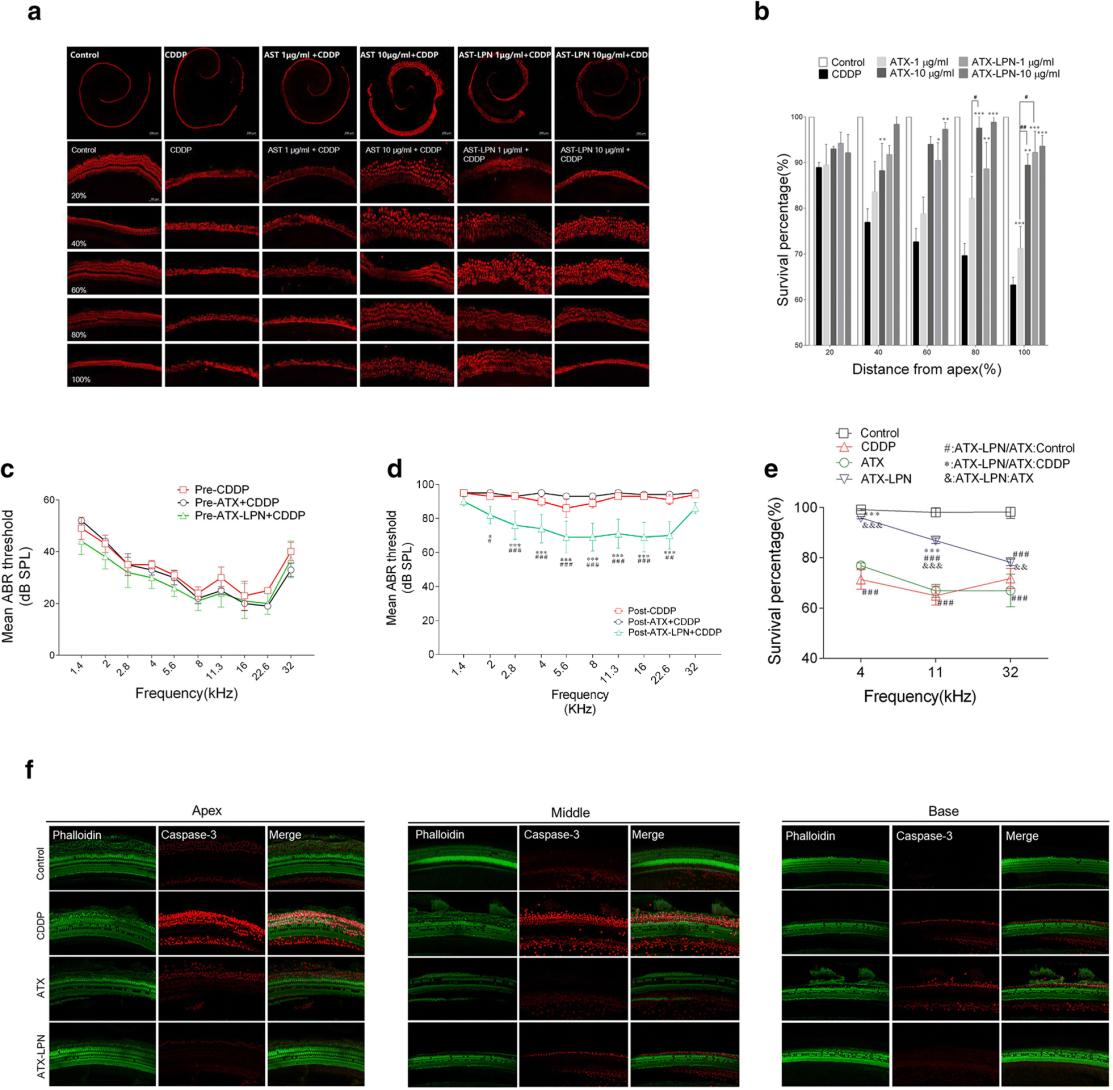
ATX-LPN partially rescue CDDP-induced hearing loss. **a** In vitro culture of Organ of Corti. CDDP induced a significant loss of hair cells (Myosin VII, red), especially in the high-frequency areas. **b** Survival outer hair cell numbers in 1 mm length at different locations from apical end of each group. *p < 0.05, **p < 0.01, ***p < 0.001 as compared with CDDP, ^#^p < 0.05 as compared with ATX + CDDP. **c**–**e** Mean ABR threshold of mice treated with cisplatin, ATX 1 mg/ml, 5 μl. ATX-LPN + CDDP. *p < 0.05, **p < 0.01, ***p < 0.001 as compared with CDDP, ^#^p < 0.05 as compared with ATX + CDDP. **f** Immunohistochemistry of cochlea in apex/middle/basal area of BM. The pretreatment of protective drugs (ATX/ATX-LPN) effectively reduced the expression of caspase-3 (red fluorescence) and rescued more OHCs (green fluorescence)
